# Qualitative Evaluation of a Clinical Decision-Support Tool for Improving Anticoagulation Control in Non-Valvular Atrial Fibrillation in Primary Care

**DOI:** 10.3390/healthcare14020199

**Published:** 2026-01-13

**Authors:** Maria Rosa Dalmau Llorca, Elisabet Castro Blanco, Zojaina Hernández Rojas, Noèlia Carrasco-Querol, Laura Medina-Perucha, Alessandra Queiroga Gonçalves, Anna Espuny Cid, José Fernández Sáez, Carina Aguilar Martín

**Affiliations:** 1 Servei Atenció Primària Terres de l’Ebre, Institut Català de la Salut, 43500 Tortosa, Tarragona, Spain; zhernandezr.ebre.ics@gencat.cat (Z.H.R.); aqueiroga@idiapjgol.info (A.Q.G.); jfernandez@idiapjgol.info (J.F.S.); caguilar.ebre.ics@gencat.cat (C.A.M.); 2 Unitat de Suport a la Recerca Terres de l’Ebre, Fundació Institut Universitari per a la Recerca a l’Atenció Primària de Salut Jordi Gol i Gurina (IDIAPJGol), 43500 Tortosa, Tarragona, Spain; ncarrasco@idiapjgol.org (N.C.-Q.); aespuny@idiapjgol.org (A.E.C.); 3 Programa de Doctorat Biomedicina, Universitat Rovira i Virgili, 43500 Tortosa, Tarragona, Spain; 4 Fundació Institut Universitari per a la Recerca a l’Atenció Primària de Salut Jordi Gol i Gurina (IDIAPJGol), 08007 Barcelona, Spain; lmedina@idiapjgol.org; 5 Universitat Autònoma de Barcelona, Bellaterra, 08193 Cerdanyola del Vallès, Barcelona, Spain; 6 Network for Research on Chronicity, Primary Care and Health Promotion (RICAPPS), 08007 Barcelona, Spain

**Keywords:** non-valvular atrial fibrillation, clinical decision-support system, primary healthcare, oral anticoagulation, time in therapeutic range, qualitative study

## Abstract

**Highlights:**

**What are the main findings?**

- Primary care professionals support a clinical decision-support system for the management of oral anticoagulation in patients with non-valvular atrial fibrillation.
- Significant barriers to clinical decision-support system adherence were identified, related to its visualization, alert fatigue, understanding of the time in therapeutic range concept, and clinical workload.

**What are the implications of the main findings?**

- To optimize the utility and adoption of the clinical decision-support system, technical improvements in its interface, better integration into the daily clinical workflow, and continuous specific training are required.
- User perceptions regarding time in therapeutic range and the system indicate the necessity to reinforce theoretical and practical knowledge in oral anticoagulation management for non-valvular atrial fibrillation to ensure informed decision-making.

**Abstract:**

**Objectives**: Clinical decision-support systems are computer-based tools to improve healthcare decision-making. However, their effectiveness depends on being positively perceived and well understood by healthcare professionals. Qualitative research is particularly valuable for exploring related behaviors and attitudes. This study aims to explore experiences of family physicians and nurses concerning the visualization, utility and understanding of the non-valvular atrial fibrillation clinical decision-support system (CDS-NVAF) tool in primary care in Catalonia, Spain. **Methods**: We performed a qualitative study, taking a pragmatic utilitarian approach, comprising focus groups with healthcare professionals from primary care centers in the intervention arm of the CDS-NVAF tool randomized clinical trial. A thematic content analysis was performed. **Results**: Thirty-three healthcare professionals participated in three focus groups. We identified three key themes: (1) barriers to tool adherence, encompassing problems related to understanding the CDS-NVAF tool, alert fatigue, and workload; (2) using the CDS-NVAF tool: differences in interpretations of Time in Therapeutic Range (TTR) assessments, and the value of TTR for assessing patient risk; (3) participants’ suggestions: improvements in workflow, technical aspects, and training in non-valvular atrial fibrillation management. **Conclusions**: Healthcare professionals endorsed a clinical decision-support system for managing oral anticoagulation in non-valvular atrial fibrillation patients in primary care. However, they emphasized the view that the CDS-NVAF requires technical changes related to its visualization and better integration in their workflow, as well as continuing training to reinforce their theoretical and practical knowledge for better TTR interpretation.

## 1. Introduction

Clinical decision-support systems are computer-based tools designed to assist healthcare decision-makers. By analyzing patient data through algorithms and by applying criteria recommended in current clinical guidelines, these systems have the potential to improve the efficiency and quality of patient care [1,2,3]. However, the success of clinical decision-support systems does not rely solely on the robustness and sophistication of their design. For these tools to provide the maximum benefits in routine clinical practice, they must also be positively perceived and well understood by healthcare professionals, who are their intended users [4,5].

Qualitative research is particularly valuable for understanding behaviors and attitudes. It is useful for co-designing new health technologies, helping to adapt them more effectively to the needs of healthcare professionals [6,7]. It may also be useful during a clinical trial, after healthcare professionals have interacted with a CDSS and gained real-world experience with the tool, as it provides insight into their views on its visualization, comprehension, and utility. By uncovering perceptions, motivations, and contextual factors, qualitative research helps identify barriers that may profoundly affect the acceptance and effectiveness of clinical decision-support systems [6,7].

In a previous randomized clinical trial conducted in public primary care centers in Catalonia, the effectiveness of the non-valvular atrial fibrillation clinical decision-support system (CDS-NVAF) was evaluated [8,9]. The tool was integrated into the electronic clinical history system of the participating centers and was designed to assist healthcare professionals during anticoagulation monitoring visits for patients with non-valvular atrial fibrillation treated with vitamin K antagonists.

Non-valvular atrial fibrillation is the most prevalent cardiac arrhythmia and is associated with increased risks of stroke, heart failure, and other complications [8]. The CDS-NVAF tool provided the Time in Therapeutic Range (TTR), calculated using the Rosendaal method, to assess anticoagulation control in patients treated with vitamin K antagonists [10]. Adequate control was defined as a TTR of ≥65% at 6 months, according to guidelines current at that time [11].

The impact of the CDS-NVAF tool was evaluated in terms of stroke, hemorrhagic events, and mortality prevention [9]. The clinical trial demonstrated reductions in both stroke and mortality rates [9]. Furthermore, the same research team examined the economic implications of poor anticoagulation control, revealing greater costs for patients with poor anticoagulation control [12].

However, before rolling out the tool to all primary care centers in Catalonia, a more thorough evaluation of user experience (nurses and family physicians) was undertaken to incorporate their input. Ensuring accurate and consistent use of the tool is critical for its success in real-world settings.

Evaluating how family physicians and nurses use the tool during routine clinical practice can provide valuable insights into its usefulness based on their experiences and interpretations. For clinical decision-support systems to genuinely increase confidence in decision-making during complex treatment choices, they must be perceived as being beneficial, non-intrusive, and allowing flexibility and control during their use [13,14]. Prioritizing workflow efficiency is also a crucial facet [15].

Additionally, it is equally important to identify areas where training or knowledge may be lacking. Training and familiarity are critical facilitators, since they increase healthcare professionals’ confidence in using the tool and improve their work efficiency [13].

Our study aimed to explore the experiences of family physicians and nurses in relation to the visualization, utility, and understanding of the CDS-NVAF tool in primary healthcare in Catalonia, Spain.

## 2. Materials and Methods

### 2.1. Study Design

We designed a qualitative descriptive study, situated within a phenomenological framework [7]. This approach sought to explore the lived experiences of healthcare professionals using the CDS-NVAF tool in real-world primary care settings in Catalonia. We adopted a pragmatic and utilitarian approach to enable a practical, useful, ethical and accurate evaluation. This approach is suitable for evaluating programmes, because it is based on the adoption of standards that require evaluations to be useful, precise, ethical and practical, rather than on the pursuit of more profound, theoretical models [16].

It consisted of focus groups in which healthcare professionals were asked about their experiences with the CDS-NVAF tool. The focus groups were selected so that participants could share, compare, and discuss their opinions and interpretations in small groups [17]. All focus groups were conducted following a standardized guide developed by the research team (Appendix A) that covered the following topics: readability and understanding of the CDS-NVAF tool’s content; usefulness of TTR monitoring during routine clinical practice, including accessibility and visibility of the monitoring documentation; opinions about (1) the responsibility for anticoagulant dosing during patient consultations, (2) as well as the effectiveness of alerts about poor anticoagulation control; clarity, use, and knowledge of the TTR value; technical difficulties in using the CDS-NVAF tool; and suggestions for improvements in the use and display of the tool.

### 2.2. Participant Recruitment

The health professionals who participated in the study were either family physicians or nurses working in the primary care system of the Catalan Health Institute, specifically from the primary care centers included in the intervention branch of the clinical trial conducted to evaluate the CDS-NVAF tool. Three focus groups were convened from different primary care centers across various regions of Catalonia. We purposively selected the centers based on geographical areas in order to obtain one representative each of a rural, an urban, and a mixed area [18].

Having selected the primary care centers, the Principal Investigator invited, via institutional email, all the family physicians and nurses from each center to participate. We sought to obtain focus groups with a minimum of six and a maximum of 12 participants. The distribution of healthcare professionals by center was as follows: 12 from Sabadell (urban area, focus group 1), 10 from Sant Vicenç de Castellet (rural area, focus group 2), and 11 participants from Tortosa (mixed urban and rural area, focus group 3). When the number of participants interested in participating in the focus group was below the maximum of 12, all eligible participants were included. When the number was greater, we purposively selected the participants to diversify the perspectives based on factors such as age, sex, professional role, and years of experience as a healthcare professional in the Catalan Institute Health. To minimize absenteeism, a reminder email was sent 48 h before each scheduled focus group session. Thirty-three healthcare professionals participated in the three focus groups: 20 nurses (2 male, 18 female) and 13 family physicians (3 male, 10 female) (Table 1).

### 2.3. Data Collection

The three focus groups, facilitated by a moderator and an observer with experience in qualitative research, were conducted between November 2019 and January 2020. Neither facilitator was involved in the development of the CDS-NVAF tool. Participants had no prior contact with any member of the research team before the focus group sessions.

Each FG lasted approximately 60 min and was digitally audio-recorded with the participants’ informed written consent. During the sessions, field notes were taken by the observer to capture group dynamics and key discussion points, which were later used to complement the audio transcripts during analysis. The audio recordings were manually transcribed verbatim by an external specialist and anonymized to ensure participant confidentiality. In the latter process, all potentially disclosive information was removed, and a unique code was assigned to each participant.

### 2.4. Data Analysis

A thematic analysis was conducted, which aimed to identify, analyze, and report patterns within the data. This process encompassed six phases: immersion in the data, generation of initial codes, identification of themes, review of the themes, definition of the final themes, and production of the final report [19]. The analysis was flexible and iterative, and, to validate the data, we practiced reflexivity during all phases of the study [20]. The three focus groups transcripts were initially coded and analyzed by the external specialist who had produced the verbatim transcripts. To enhance validity, two researchers independently double-coded a subset of transcripts. Triangulation was carried out by three members of the research team and included the analysis performed by the two researchers and the external specialist. Discrepancies were resolved through reflexive consensus. The final sample size was determined pragmatically to ensure thematic sufficiency across settings, being guided by the concept of information power [21], and considering the absence of new emerging codes (code saturation) in the last focus group analyzed.

No specialized software was needed to manage the data. Variations in data organization and theme development were examined, discussed, and reconciled to reach a consensus on the key themes. Lastly, interpretations were synthesized and illustrative quotations were included to highlight the most pertinent findings. The present study followed the trustworthiness criteria defined by Lincoln and Guba [22] as well as Korstjens and Moser [23], and it is reported in accordance with the Standards for Reporting Qualitative Research [24] (Supplementary Material S1). Ethical approval was granted by the Research Ethics Committee of IDIAPJGol (number: 19/180-P) on 30 October 2019.

### 2.5. The Tool

The CDS-NVAF tool is a system integrated within the electronic clinical history database of primary care centers in Catalonia. Its purpose is to assist healthcare professionals during anticoagulation monitoring visits of patients with non-valvular atrial fibrillation treated with vitamin K antagonists.

Anticoagulation therapy is crucial for non-valvular atrial fibrillation patients, because maintaining international normalized ratio (INR) values within the therapeutic range of 2–3 for ≥65% of the time over a period of 6 months, as suggested by Rosendaal [10], balances the risks of stroke and bleeding. To provide a more comprehensive view of the patient’s anticoagulation management, the CDS-NVAF tool calculates the TTR using the Rosendaal method [10]. This assesses the degree of anticoagulation control based on INR values from the past 6 months, expressed as a percentage. The tool sets the threshold for adequate anticoagulation control at a TTR of ≥65%, while a TTR of <65% indicates poor anticoagulation control [8,25]. This ≥65% target follows locally harmonized clinical guidance, and the CDS-NVAF pop-up alert is triggered when the 6-month Rosendaal TTR falls below this threshold.

When a new INR value is entered, the CDS-NVAF automatically recalculates the TTR. The CDS-NVAF tool displays the TTR percentage on the electronic clinical history screen. Previous INR and TTR values remain visible on the anticoagulant follow-up screen within the electronic clinical history, allowing for easy reference during future consultations. For patients with poor anticoagulation control (TTR < 65%), this percentage is highlighted in a distinct color to draw attention to it immediately. Additionally, when a patient has a TTR of <65% from the past 6 months, the tool alerts the healthcare professional responsible for vitamin K antagonist monitoring (usually a nurse). The alert appears as a pop-up notification and suggests that a switch to direct oral anticoagulants be considered.

Before the CDS-NVAF tool was incorporated into the electronic clinical history system, healthcare professionals used to calculate manually the number of INR values within the therapeutic range over the previous 6 months by the direct method, which only considered INR values within the range of 2 to 3 from the previous 6 months and set the threshold for adequate anticoagulation control at 60% [26].

Healthcare professionals were informed about the tool and its functionality through two informational notes at the beginning of the clinical trial. This is the standard procedure when new components are incorporated in the electronic clinical history system, so the dissemination of the CDS-NVAF tool followed standard clinical practice. These notes included an announcement displayed upon opening the electronic clinical history system on the first day of the intervention that provided access to the CDS-NVAF tool [8].

## 3. Results

Thirty-three healthcare professionals, including 20 nurses and 13 family physicians (28 women and 5 men), participated in the three focus groups. Their median age was 53 years. They had a median of 30 years of work experience (Table 1).

Three key themes emerged from the focus groups and interviews. The first centered on the challenges participants encountered to their compliance with using the tool. It included subthemes related to the comprehension of the CDS-NVAF tool, alert fatigue and workload. The second theme concerned aspects related to the experience of using the tool. Subthemes included technical aspects related to access and usability, as well as experiences in clinical practice with the tool and its utility. The third theme covered participants’ suggestions for improving the tool, forming three subthemes: improved workflow, technical improvements, and training in the management of non-valvular atrial fibrillation (Table 2).

### 3.1. Theme 1: Challenges to Compliance with Using the Tool

#### 3.1.1. Comprehension of the Tool

The professionals generally considered the CDS-NVAF tool to be understandable. However, some participants admitted they did not fully understand the meaning of the different TTR values on the patient anticoagulant follow-up screen. As a result, they also expressed some uncertainty about the purpose of the alert, questioning whether it is triggered by a single out-of-range value or if it indicates a persistent issue. Such uncertainty is a key issue for clinical decision-making.

Some participants admitted unfamiliarity with the presence of the CDS-NVAF tool in the electronic clinical history and preferred to rely on manual calculations with previous INR values and to apply their own criteria based on their professional knowledge.

*“No, no, I didn’t know about the TTR… And, honestly, we work based on common sense… you know… let’s see… you keep an eye on adherence, check the range to see if it’s in, the INR, and when you see that it doesn’t quite add up, you calculate it and so on, and you discuss it with the medical team.”* (woman, nurse, focus group 2)

#### 3.1.2. Alert Fatigue and Workload

One challenge affecting tool compliance is how alerts in the electronic clinical history are managed. Some focus group participants, especially family physicians, reported being overwhelmed by the excessive number of alerts received from the electronic clinical history arising from various health issues, which made it difficult to manage the health conditions effectively. They also cited a lack of time and workload pressure as reasons for not reviewing all the valuable information contained in alerts as thoroughly as they would have liked, which impacted the coordination of patient care.

*“We have to look at so many things… That…, honestly, I’m so tired of it, you know… I do it because it has to be done, but with the patient load we have…, I mean…”* (woman, family physician, focus group 3)

### 3.2. Theme 2: Using the CDS-NVAF Tool

#### 3.2.1. Technical Issues: Access and Usability

The vast majority of healthcare professionals accessed the CDS-NVAF tool either as instructed during a training session spontaneously offered by managers of their primary care center, or as explained by their co-workers.

Some participants expressed their frustration with the new system because it did not allow manual changes to be saved or consistently applied to all subsequent weeks. This prompted them to request that the alerts were adapted accordingly to patients’ circumstances (temporary treatment suspension, dose changes, changes in control frequency, among others).

*“The diagnoses and all that… I don’t know… something should be done so it’s reflected somehow that there’s… so you don’t have to go digging through medical records to figure out what the problem is… Because if it were right there with the diagnosis, saying he has valvulopathy and a valve prosthesis… you’d already know you need to keep it at 2.5… you’d see it in the diagnosis and maybe we wouldn’t make the mistake of setting it too low or… I don’t know, really. It’s just that…”* (woman, nurse, focus group 1)

Simultaneously, some participants noted that the CDS-NVAF tool allowed them to simply click *Accept* and continue without receiving any further guidance on addressing the issue. Consequently, they criticized the alert function for “encouraging” them to ignore it, allowing them to proceed with patient monitoring while potentially missing critical cases of altered values.

#### 3.2.2. Clinical Practice with the Tool

Nurses noted that the alert appeared only to them, not to family physicians, during TTR control consultations, but they were not permitted to manage it. Alerts could be handled appropriately only by family physicians, after being informed by nurses. This disrupted the schedules of both.

*“When it comes to Sintrom (vitamin K antagonist medication)… you get the alert, and it doesn’t let the nurse… it doesn’t let her handle the Sintrom… and then you have to pass it on to the doctor colleague …”* (woman, nurse, focus group 3)

Predominantly, participants reported having incorporated the CDS-NVAF tool into their routine clinical practice. However, different healthcare professionals interpreted TTR assessments differently. Some generally considered them a wake-up call but not something necessarily requiring intervention because they had to evaluate each case individually anyway in order to make clinical decisions.

*“When I get the alarm (TTR alert) for the first time, I verify everything and focus on the individual patient. But if the alarm comes up a second time, whether I know the patient or not, I bring it to the doctor’s attention, because a second time means… Maybe not with the first alarm, because first you need to screen to see what’s happening, since it could be that… well, analyse what’s going on. But if the alarm triggers again at the next check… it’s not being… at some point you have to.”* (woman, family physician, focus group 2)

Others reported using large INR fluctuations between control assessments as the main sign of poor TTR management. This led them to pay more attention and review such cases when they arose, or to conduct an extensive review when an alert was triggered, especially for a second time. This usually resulted in a change to the treatment plan.

*“Well, if it’s been showing highs and lows for many months (INR values), I assume that means if it’s been going on for months, the levels are out of range. This is a poorly controlled patient, right?”* (woman, nurse, focus group 1)

#### 3.2.3. Utility of the Tool

In all three focus groups, participants recognized the importance of TTR in assessing patient anticoagulation control. Predominantly, participants found the alert useful, especially for detecting potential medication issues. They noted its value for identifying TTR fluctuations and investigating their causes, such as missing a dose or taking other treatments that affect TTR assessments.

*“What? The alert? Oh, yeah…!!! For me… Yes, yes, yes, of course! Because it warns you, right? That something’s off… If the range isn’t good, if it’s not at the levels it shows there, well something’s wrong, and we need to check, right? Why…”* (woman, family physician, focus group 1)

However, they emphasized that although the tool provides valuable information, family physicians are responsible for the final clinical decision-making.

*“With screenings… Because for example, when I’m handling other people’s patients and the alarm (TTR alert) goes off, I have to check the clinical course, maybe this alarm was already assessed. That’s when you say ‘Okay, this was already sent and I see the doctor noted here they won’t change the treatment…’ And you bring it up again and they’re like ‘no no, I already assessed that…’ so you say alright then… and that’s that, but you still record it…”* (woman, nurse, focus group 2)

### 3.3. Theme 3: Participants’ Suggestions

#### 3.3.1. Better Workflow

The participants suggested that nurses, in addition to family physicians, should be allowed to manage CDS-NVAF alerts directly, with family physicians being automatically notified. This would reduce interruptions and improve workflow efficiency.

*“It could also show up for the doctor… Because sometimes you have a low TTR, but sometimes you don’t have enough information, or you’re in such a hurry that you don’t notice that there’s a 30% therapeutic time where you might need to change the treatment… So, it could also show up for you…”* (woman, family physician, focus group 3)

#### 3.3.2. Technical Improvements

According to participants’ opinion, the electronic clinical history system should be simplified to increase alert productivity. At the same time, the system should allow manual adjustments and annotations about patient-specific circumstances affecting treatment in the anticoagulant spreadsheet. The spreadsheet should also be linked to patients’ overall health records, especially for patients with poor adherence to anticoagulant therapy or for those with other factors affecting TTR assessments.

*“Maybe…, for example, if you need to change the… anticoagulation therapy, from oral anticoagulants to low molecular weight heparin, that there would be some place where it could be recorded… For example, if you have to stop Sintrom (vitamin K antagonists medication), it could already say, ‘The patient has surgery… three days before, Sintrom should be stopped.’ So, on the same spreadsheet, it could show… an injection that needs to be administered, when to stop Sintrom, and when to restart it…”* (woman, nurse, focus group 1)

#### 3.3.3. Training

Participants suggested the need for ongoing training regarding NVAF management and anticoagulation control, including the different meanings and implications of TTR, as they found that the currently available training courses are not useful or effective in enabling proper patient follow-up.

They insisted that theoretical training on non-valvular atrial fibrillation and TTR management, as well as practical workshops on the operation of digital tools in electronic clinical history system, should be introduced. This would raise awareness of the importance of the TTR assessment and the regrettable current underuse of the CDS-NVAF tool. The professionals highlighted the importance of new healthcare professionals receiving training in non-valvular atrial fibrillation and the CDS-NVAF tool, preferably before starting work in patient healthcare.

*“We talk about this a lot and… well, we think that before joining the ICS (Catalan Health Institute), before starting to work at a primary care centre, there should be a course… for nurses, for doctors…, where they teach you everything about eCAP (electronic clinical history system). Properly. It would be fantastic because when you get here… And eCAP is a good tool. This is a bit… a request… That people who are on the waiting list, close to being hired, should already take this course…”* (woman, nurse, focus group 1)

The most representative verbatim quotations for each subtheme are provided in Supplementary Material S2.

## 4. Discussion

In the present study we found that the tool was useful in providing support by reinforcing healthcare professionals’ decision-making by emphasizing the need to improve anticoagulation control, either through better adherence to vitamin K antagonist medication or by switching to a different anticoagulant, and by promoting teamwork. However, feedback from the study participants revealed that some healthcare professionals at primary care centers in Catalonia who were using the CDS-NVAF tool did not fully understand it and believed they required additional training. They were also clear that it needs improvements to prevent it from interfering with their workflow.

The potential benefits of the CDS-NVAF tool may have been undermined by the lack of clarity regarding the meaning of the TTR percentage, along with workload pressure and systemic alert fatigue within the electronic clinical history. This often leads to alerts being unnoticed or ignored, which prevents further action being taken. Other studies employing clinical decision-support systems for atrial fibrillation have encountered comparable barriers and limitations regarding excessive alerts in the electronic clinical history, which often resulted in the alerts being ignored [14,27]. They also reported low adoption rates of the alert recommendations, which could be attributed to time constraints and additional workload, alert fatigue from excessive notifications and system malfunctions, all of these arising from the poor adaptability of these tools to clinical workflows [14,27]. As a result of these limitations, other tools used for improving non-valvular atrial fibrillation management were generally unable to demonstrate better efficacy than that provided by usual care, nor did they lead to a reduction in stroke or better anticoagulation control or adherence [2,27,28,29]. However, the CDS-NVAF tool has been shown to be modestly but significantly effective in reducing stroke and mortality [9].

While access to the vitamin K antagonist anticoagulation control assessment page in the electronic clinical history was shown to be consistent in the present study, healthcare professionals have reported several challenges. These include difficulties with automatic access, the inability to save manual changes on the CDS-NVAF tool, and challenges for nurses in handling CDS-NVAF alerts properly, including the additional responsibility of notifying family physicians about them. Together, these issues could create additional burdens during anticoagulation control visits. Furthermore, technical issues have been reported, which limit the usefulness of the tool and increase the workload. Such integration barriers to clinical workflows across healthcare professional roles not only increase workload but also undermine the potential utility of the clinical decision-support systems [15,27]. This led some participants to express a preference for manual assessments. All these matters have raised concerns among some healthcare professionals regarding the robustness and reliability of the tool [15].

The most pressing issue is that some healthcare professionals do not fully understand the TTR assessment procedure, which highlights the need for informative sessions and training. Information was only provided before the clinical trial assessing the effectiveness and impact of the CDS-NVAF tool when healthcare professionals were informed about its functionality through two informational notes. These included an announcement displayed when opening the electronic clinical history on the first day of the intervention, which provided access to the CDS-NVAF tool and explained its functionality and clinical significance [8].

The CDS-NVAF tool triggers an alert during anticoagulation control visits, when nurses are responsible for INR monitoring. The alert appears as a pop-up notification on the electronic clinical history screen and is primarily seen by nurses when new INR values are entered and the TTR is automatically recalculated. It indicates that the patient has poor anticoagulation control (TTR < 65% over the previous six months) and advises reviewing the current anticoagulation treatment and considering a switch to direct oral anticoagulants, a decision for which only family physicians are responsible (Supplementary Material S3).

During the initial pilot testing phase, when alerts identified patients with poor anticoagulation control, the system prevented nurses from continuing their visits, forcing them to interrupt family physicians immediately, which was criticized by both groups of professionals. To address this disruption, the functionality was modified during the clinical trial by introducing an *Accept* button that allowed nurses to acknowledge alerts and proceed with their work during visits. However, this solution also raised concerns, as some felt the tool “encouraged” them to dismiss alerts without taking further corrective actions, and nurses still had to inform family physicians.

Therefore, some participants suggested that nurses should be permitted to handle alerts directly, with family physicians being notified automatically. Although nurses are not authorized to prescribe, adjust, or discontinue patients’ anticoagulation medication by themselves, this responsibility being discharged solely by family physicians, they could receive support from the tool through on-screen tips or built-in guides that explain the steps that must be taken when an alert is triggered.

According to the literature, the successful implementation of clinical decision-support systems into routine clinical practice is largely determined by the professionals’ attitudes [4,5]. The availability of electronic clinical history has further enabled the use of clinical decision-support systems, providing healthcare professionals with individualized, evidence-based support [3,8,30,31,32]. However, some family physicians reported that clinical decision-support systems are designed without considering their perspective, and so do not adapt them to fit their workflow [13]. In this regard, co-design between researchers and tool developers would be advantageous, particularly with respect to the tool’s visualization. In public healthcare systems, the tools and the ways they are implemented are the sources of the limitations inherent to their public administration.

Three distinct profiles emerge from the analysis of family physicians’ perspectives: the skeptical family physician, who perceives clinical decision-support systems as an additional burden; the theoretical FP, who acknowledges the potential benefits of clinical decision-support systems but doubts their practical efficacy; and the advocate family physician, who sees clinical decision-support systems as a valuable tool for decision-making support [4]. Our qualitative analysis revealed a high proportion of those with skeptical profiles and of theoretical professionals who acknowledged the tool’s benefits but appeared to not fully understand its functionality and clinical significance. This finding is in keeping with previous studies of clinical decision-support systems for rhythm management in patients with atrial fibrillation, in which family physicians expressed several concerns. Their main focus was on the safety of the recommendations provided by the system and the clinical reasoning behind them because they needed to ensure their recommendations made sense in practice [33]. This desire was particularly strong in senior family physicians, who demanded total transparency about how the clinical decision-support systems operated and wanted assurance that they were linked to appropriate evidence [34]. They also emphasized the importance of having the flexibility to challenge decisions made by the system and to interpret outputs, since these recommendations are often perceived as prescriptive rather than supportive [13,33].

In contrast, studies of a clinical decision-support system for atrial fibrillation implemented with pharmacists showed higher acceptance rates [35]. Consistent with the findings from the family physicians of the primary care centers in our study, the pharmacists also reported limited knowledge about atrial fibrillation and drew attention to their training needs. The family physicians also expressed a desire for additional technological tools that could provide more effective support [36].

During the present study, significant educational gaps concerning the purpose and management of TTR assessments were identified. Despite implementing a potentially useful tool, the lack of training on TTR has resulted in it not always being perceived as useful [13,33]. The preference of many healthcare professionals for manual calculations denotes a lack of confidence in the tool’s reliability or a lack of training in how to interpret its outputs. Even when perceived as relevant, the tool is not always used correctly. Moreover, the fact that some healthcare professionals considered the alert to be merely a wake-up call that does not necessarily require intervention, while others rely on fluctuations in assessments as the main indicator of poor anticoagulation management, highlights the need for training in the tool’s functionality and in anticoagulation management.

To address these issues, a large number of participants advocated for the implementation of ongoing training programmes for healthcare professionals involved in vitamin K antagonists monitoring [35,36]. These programmes should ensure theoretical and practical understanding of TTR values, provide hands-on training with the CDS-NVAF tool, and clarify the significance of alerts and the appropriate responses to them [13].

Beyond clinical effectiveness, the cost-effectiveness of implementing clinical decision-support systems is a critical factor for healthcare organizations. Recent evidence suggests that such tools optimize resource allocation by reducing costs and improving electronic document management, making them economically sustainable in the long term [37]. However, the successful integration of these systems is inextricably linked to continuous professional development. To ensure long-term sustainability and overcome implementation barriers, healthcare systems must prioritize ongoing technical and theoretical training. This ensures that clinicians remain proficient not only in the evolving functionalities of electronic health records but also in the latest clinical guideline updates, ultimately fostering higher adherence to the tool.

The CDS-NVAF tool, like other clinical decision-support systems, is based on evidence [10], current guidelines, clinical pathways, and algorithms, and aims to enable individualized and timely treatment. It facilitates decision-making but does not replace or override the role of healthcare professionals in making appropriate clinical decisions [38].

### Limitations

This study has several limitations regarding its representativeness and temporal framework. Although the study covered urban, rural, and mixed primary care settings, the qualitative phase was conducted in only three centers within the Catalan Health Institute (ICS) between November 2019 and January 2020. Consequently, the findings reflect a specific public primary care context and may not fully encompass the diversity of professional perspectives across other regions. Furthermore, the technological evolution of the electronic clinical history, updates in clinical alert policies, and the significant increase in the adoption of direct oral anticoagulants since the focus groups were conducted may influence current perceptions of the tool. Finally, there was a predominance of female participants; however, this gender imbalance is a frequent characteristic of studies conducted in Catalonia and reflects the actual demographic distribution of healthcare professionals in the region.

## 5. Conclusions

The CDS-NVAF tool can be useful in providing support by reinforcing healthcare professionals’ decision-making, emphasizing the need to assess adherence to vitamin K antagonists or to change anticoagulant therapy, and promoting teamwork.

Participants commented that the access and usability of the CDS-NVAF tool could have been affected by the systematic alert fatigue caused by the use of the electronic clinical history system, workload pressure, and technical issues. Their feedback highlighted the need for technical changes, workflow improvements, a better understanding of the tool, and additional training.

## Figures and Tables

**Table 1:** Frequencies of participants by focus group and healthcare profession.

Focus Group	Focus Group 1	Focus Group 2	Focus Group 3	Total
Sociocultural Context	Urban Area	Rural Area	Mixed Area	
n	12 (36.36%)	10 (30.30%)	11 (33.33%)	33 (100%)
Family physicians	5 (15.15%)	3 (9.09%)	5 (15.15%)	13 (39.39%)
Female	4 (12.12%)	3 (9.09%)	3 (9.09%)	10 (30.30%)
Nurses	7 (21.21%)	7 (21.21%)	6 (18.18%)	20 (60.61%)
Female	7 (21.21%)	5 (15.15%)	6 (18.18%)	18 (54.55%)
Age of all participants (years)				
Median (IQR)	53 (13.5)			
Work experience of all participants (years)				
Median (IQR)	30 (17.5)			

IQR: interquartile range.

**Table 2:** Themes and subthemes emerging from the focus groups regarding the visualization, utility and understanding of the non-valvular atrial fibrillation clinical decision-support system (CDS-NVAF) tool.

Themes	Subthemes
Challenges to compliance with using the tool	Comprehension of the tool
	Alert fatigue and workload
Using the CDS-NVAF tool	Technical issues: access and usability
	Clinical practice with the tool
	Utility of the tool
Participants’ suggestions	Better workflow
	Technical improvements
	Training

## Data Availability

The data presented in this study are available on request from the corresponding author due to privacy or ethical restrictions.

## Supplementary Materials

The following supporting information can be downloaded at https://www.mdpi.com/article/10.3390/healthcare14020199/s1. Material S1: Standards for Reporting Qualitative Research (SRQR). Material S2: Relation of verbatims and subthemes. Material S3: Alert of CDS-NVAF triggered during anticoagulation control visits.

## Abbreviations

The following abbreviations are used in this manuscript:

CDS-NVAF	Non-valvular atrial fibrillation clinical decision-support system
TTR	Time in Therapeutic Range

## Appendix A. Focus Group Topics

- User feedback on the usefulness and limitations of the informative notes on the electronic clinical history system when the TTR monitoring tool was installed.
  1. How do you usually access the anticoagulant dosing sheet: through ‘Other sheets’ or ‘Oral anticoagulation monitoring’?
  2. Who is responsible for anticoagulant dosing for patients during consultations: Family physicians or nurses?
  3. Have you ever noticed the alert that warns about poor TTR control, indicating that adjusting medication is imperative? Is the alert visible enough? Is its message understandable?
  4. Is the ‘Oral anticoagulation monitoring’ sheet visible enough?
  5. Is it clear why TTR may not be assessed at times?
  6. Have you ever consulted the ‘i’ icon located next to the variable to understand how the TTR value is assessed at 6, 12 and 24 months?
  7. Do you know what TTR value is considered indicative of poor anticoagulation control, warranting a switch in the oral anticoagulant?
- Problems using the TTR monitoring tool.
  1. Which technical difficulties have you experienced when using the TTR monitoring tool?
- Improvements in the use and display of the TTR monitoring tool.
  1. Can you suggest some improvements to the use and display of the TTR monitoring tool?
