# Supplementary material for: Qualitative Evaluation of a Clinical Decision-Support Tool for Improving Anticoagulation Control in Non-Valvular Atrial Fibrillation in Primary Care
Source: Healthcare (Basel). 2026 Jan 13;14(2):199. doi: 10.3390/healthcare14020199 (PMC12840853; doi:10.3390/healthcare14020199)
Supplement: Supplementary file 1 [file healthcare-14-00199-s001.zip › healthcare-4038419-supplementary.pdf]

Supplementary Material S1: Standards for Reporting Qualitative Research (SRQR)

http://www.equator-network.org/reporting-guidelines/srqr/

Title and abstract

	Page/line no(s).
**Title** - Concise description of the nature and topic of the study Identifying the study as qualitative or indicating the approach (e.g., ethnography, grounded theory) or data collection methods (e.g., interview, focus group) is recommended	1/2-4
**Abstract** - Summary of key elements of the study using the abstract format of the intended publication; typically includes background, purpose, methods, results, and conclusions	1-2/35-57

Introduction

**Problem formulation** - Description and significance of the problem/phenomenon studied; review of relevant theory and empirical work; problem statement	2-3/62-111
**Purpose or research question** - Purpose of the study and specific objectives or questions	3/109-111

Methods

**Qualitative approach and research paradigm** - Qualitative approach (e.g., ethnography, grounded theory, case study, phenomenology, narrative research) and guiding theory if appropriate; identifying the research paradigm (e.g., postpositivist, constructivist/ interpretivist) is also recommended; rationale**	3/114-120
**Researcher characteristics and reflexivity** - Researchers' characteristics that may influence the research, including personal attributes, qualifications/experience, relationship with participants, assumptions, and/or presuppositions; potential or actual interaction between researchers' characteristics and the research questions, approach, methods, results, and/or transferability	4/155-158
**Context** - Setting/site and salient contextual factors; rationale**	3-4/133-139
**Sampling strategy** - How and why research participants, documents, or events were selected; criteria for deciding when no further sampling was necessary (e.g., sampling saturation); rationale**	3-4/133-139
**Ethical issues pertaining to human subjects** - Documentation of approval by an appropriate ethics review board and participant consent, or explanation for lack thereof; other confidentiality and data security issues	4/159-160<br>4/162-165<br>5/187-188<br>12/542-546
**Data collection methods** - Types of data collected; details of data collection procedures including (as appropriate) start and stop dates of data collection and analysis, iterative process, triangulation of sources/methods, and modification of procedures in response to evolving study findings; rationale**	4/155-165

**Data collection instruments and technologies** - Description of instruments (e.g., interview guides, questionnaires) and devices (e.g., audio recorders) used for data collection; if/how the instrument(s) changed over the course of the study	3/122-131<br>4/155-165
**Units of study** - Number and relevant characteristics of participants, documents, or events included in the study; level of participation (could be reported in results)	4/143-145<br>5/226-228
**Data processing** - Methods for processing data prior to and during analysis, including transcription, data entry, data management and security, verification of data integrity, data coding, and anonymization/de-identification of excerpts	4/159-165
**Data analysis** - Process by which inferences, themes, etc., were identified and developed, including the researchers involved in data analysis; usually references a specific paradigm or approach; rationale**	4-5/167-188
**Techniques to enhance trustworthiness** - Techniques to enhance trustworthiness and credibility of data analysis (e.g., member checking, audit trail, triangulation); rationale**	4/173-180

Results/findings

**Synthesis and interpretation** - Main findings (e.g., interpretations, inferences, and themes); might include development of a theory or model, or integration with prior research or theory	6/233-239
**Links to empirical data** - Evidence (e.g., quotes, field notes, text excerpts, photographs) to substantiate analytic findings	6-9/244-375

Discussion

**Integration with prior work, implications, transferability, and contribution(s) to the field -** Short summary of main findings; explanation of how findings and conclusions connect to, support, elaborate on, or challenge conclusions of earlier scholarship; discussion of scope of application/generalizability; identification of unique contribution(s) to scholarship in a discipline or field	9-11/376-505
**Limitations** - Trustworthiness and limitations of findings	11-12/506-518

Other

**Conflicts of interest** - Potential sources of influence or perceived influence on study conduct and conclusions; how these were managed	12-13/554-561
**Funding** - Sources of funding and other support; role of funders in data collection, interpretation, and reporting	12/537-541

*The authors created the SRQR by searching the literature to identify guidelines, reporting standards, and critical appraisal criteria for qualitative research; reviewing the reference lists of retrieved sources; and contacting experts to gain feedback. The SRQR aims to improve the transparency of all aspects of qualitative research by providing clear standards for reporting qualitative research.

**The rationale should briefly discuss the justification for choosing that theory, approach, method, or technique rather than other options available, the assumptions and limitations implicit in those choices, and how those choices influence study conclusions and transferability. As appropriate, the rationale for several items might be discussed together.

Reference: O'Brien BC, Harris IB, Beckman TJ, Reed DA, Cook DA. **Standards for reporting qualitative research: a synthesis of recommendations.** *Academic Medicine,* Vol. 89, No. 9 / Sept 2014 DOI: 10.1097/ACM.0000000000000388

Supplementary Material S2: Relation of verbatims and subthemes.

Subthemes	
Comprehension of the tool	*"No, no, I didn't know about the TTR... And, honestly, we work based on common sense… you know... let's see... you keep an eye on adherence, check the range to see if it's in, the INR, and when you see that it doesn't quite add up, you calculate it and so on, and you discuss it with the medical team." (woman, nurse, FG 2)*
Alert fatigue and workload	*"We have to look at so many things...That..., honestly, I'm so tired of it, you know… I do it because it has to be done, but with the patient load we have..., I mean..."* (woman*, family physician, FG 3)*
Technical issues: access and usability	*"The diagnoses and all that… I don't know… something should be done so it's reflected somehow that there's… so you don't have to go digging through medical records to figure out what the problem is… Because if it were right there with the diagnosis, saying he has valvulopathy and a valve prosthesis… you'd already know you need to keep it at 2.5… you'd see it in the diagnosis and maybe we wouldn't make the mistake of setting it too low or… I don't know, really. It's just that…" (woman, nurse, FG 1)*
Clinical practice with the tool	*"When I get the alarm (TTR alert) for the first time, I verify everything and focus on the individual patient. But if the alarm comes up a second time, whether I know the patient or not, I bring it to the doctor's attention, because a second time means... Maybe not with the first alarm, because first you need to screen to see what's happening, since it could be that... well, analyse what's going on. But if the alarm triggers again at the next check... it's not being... at some point you have to." (woman, family physician, FG 2)*
Utility of the tool	*"What? The alert? Oh, yeah...!!! For me... Yes, yes, yes, of course! Because it warns you, right? That something's off... If the range isn't good, if it's not at the levels it shows there, well something's wrong, and we need to check, right? Why..." (woman, family physician, FG 1)*
Better workflow	*"It could also show up for the doctor… Because sometimes you have a low TTR, but sometimes you don't have enough information, or you're in such a hurry that you don't notice that there's a 30% therapeutic time where you might need to change the treatment… So, it could also show up for you…" (woman, family physician, FG 3)*
Technical improvements	*"Maybe…, for example, if you need to change the… anticoagulation therapy, from oral anticoagulants to low molecular weight heparin, that there would be some place where it could be recorded… For example, if you have to stop Sintrom (VKA medication), it could already say, 'The patient has surgery… three days before, Sintrom should be stopped.' So, on the same spreadsheet, it could show… an injection that needs to be administered, when to stop Sintrom, and when to restart it…" (woman, nurse, FG 1)*

Training	*"We talk about this a lot and… well, we think that before joining the ICS (Catalan Health Institute), before starting to work at a primary care centre, there should be a course… for nurses, for doctors…, where they teach you everything about eCAP (ECH system). Properly. It would be fantastic because when you get here... And eCAP is a good tool. This is a bit… a request… That people who are on the waiting list, close to being hired, should already take this course..." (woman, nurse, FG 1).*

Supplementary Material S3: Alert of CDS-NVAF triggered during anticoagulation control visits.

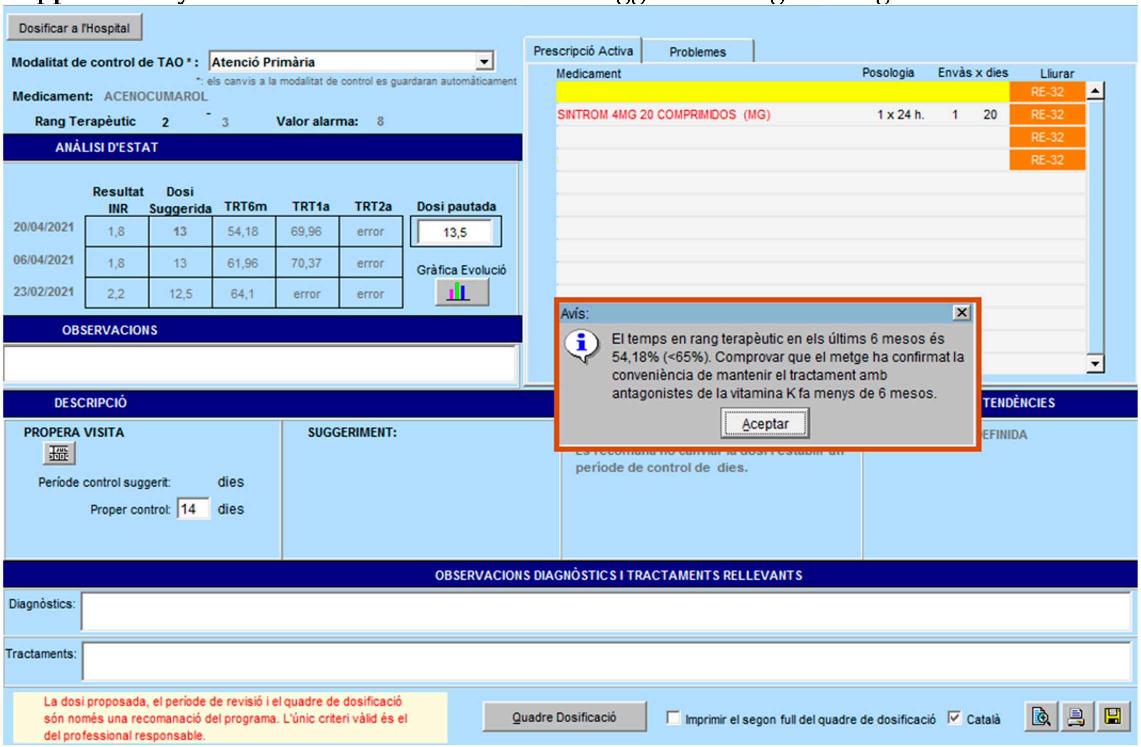

Alert: The time in therapeutic range in the last 6 months is 54.18% Check that the doctor has confirmed the advisability of maintaining the treatment with vitamin K antagonists less than 6 months ago.
